# Real-World Management of High-Risk Prostate Cancer Post-Radical Prostatectomy: Insights from a Regional Quality Collaborative

**DOI:** 10.3390/cancers17101600

**Published:** 2025-05-08

**Authors:** Aaron R. Hochberg, Annie H. Ho, Rasheed A. M. Thompson, Matthew B. Buck, Costas D. Lallas, Christine Ibilibor, Jeffrey J. Tomaszewski, Serge Ginzburg, Andres Correa, Robert Uzzo, Marc C. Smaldone, John F. Danella, Thomas J. Guzzo, Daniel J. Lee, Laurence Belkoff, Jeffrey Walker, Jay D. Raman, Roderick K. Clark, Adam Reese, Bruce Jacobs, Thomas Jang, Keith J. Kowalczyk, Meghan Smith, Mihir S. Shah

**Affiliations:** 1 Department of Urology, Sidney Kimmel Medical College at Thomas Jefferson University, Philadelphia, PA 19107, USA; aaron.hochberg@students.jefferson.edu (A.R.H.); annie.ho@students.jefferson.edu (A.H.H.); rasheed.thompson@jefferson.edu (R.A.M.T.); matthew.buck@jefferson.edu (M.B.B.); costas.lallas@jefferson.edu (C.D.L.); 2 Department of Urology, Howard University College of Medicine, Washington, DC 20059, USA; 3 Department of Urology, University of Virginia, Charlottesville, VA 22904, USA; ci5d@uvahealth.org; 4 Division of Urology, Cooper University Health Care, Camden, NJ 08103, USA; tomaszewski-jeffrey@cooperhealth.edu; 5 Department of Urology, Einstein Healthcare Network, Philadelphia, PA 19141, USA; serge.ginzburg@jefferson.edu; 6 Division of Urologic Oncology, Fox Chase Cancer Center, Philadelphia, PA 19111, USA; andres.correa@fccc.edu (A.C.); robert.uzzo@fccc.edu (R.U.); marc.smaldone@fccc.edu (M.C.S.); 7 Department of Urology, Geisinger Health System, Danville, PA 17822, USA; jdanella@geisinger.edu; 8 Division of Urology, University of Pennsylvania, Philadelphia, PA 19104, USA; thomas.guzzo@pennmedicine.upenn.edu (T.J.G.); daniel.lee4@pennmedicine.upenn.edu (D.J.L.); 9 Division of Urology, MidLantic Urology, Main Line Health, Bala Cynwyd, PA 19010, USA; lbelkoff@midlanticurology.com (L.B.); jwalker@midlanticurology.com (J.W.); 10 Department of Urology, Penn State Health, Hershey, PA 17033, USA; jraman@pennstatehealth.psu.edu (J.D.R.);; 11 Department of Urology, Temple University, Philadelphia, PA 19122, USA; adam.reese@tuhs.temple.edu; 12 Department of Urology, University of Pittsburgh Medical Center, Pittsburgh, PA 15261, USA; jacobsbl2@upmc.edu; 13 Division of Urology, Rutgers Cancer Institute of New Jersey, New Brunswick, NJ 08901, USA; jangtl@cinj.rutgers.edu; 14 Department of Urology, MedStar Health Georgetown, Washington, DC 20007, USA; keith.kowalczyk@medstar.net; 15 Health Care Improvement Foundation, Philadelphia, PA 19103, USA; msmith@hcifonline.org

**Keywords:** prostate cancer, adjuvant therapy, salvage therapy, lymph node metastasis, disease management

## Abstract

**Background/Objectives**: Data from clinical trials showing the non-inferiority of early salvage radiotherapy were recently incorporated into societal guideline statements. However, questions remain regarding ideal prostate-specific antigen (PSA) criteria for salvage, and how to apply these findings to pathologic lymph node-positive (pN+) disease. We investigated variance in management of clinically localized prostate cancer found to have high-risk features after radical prostatectomy. **Methods**: We retrospectively identified patients from May 2015 to January 2024 utilizing a multi-institutional, regional collaborative database. The primary outcome was identifying factors associated with the receipt of adjuvant versus salvage therapy. Factors associated with secondary treatment were assessed via multivariable logistic regression. **Results**: In total, 230 (38%) patients received adjuvant and 375 (62%) received salvage therapy. Rates of adjuvant versus salvage therapy differed by practice setting (*p* < 0.001). A higher percentage of patients received salvage (38.9%) over adjuvant (13.5%) therapy in or after 2020 (*p* < 0.001). In our model, patients with preoperative PSA ≥ 10 ng/mL (OR: 2.15, CI: 1.31–3.53) and treatment in or after 2020 (OR: 3.41, CI: 1.75–6.66) had higher odds, while patients with persistent detectable postoperative PSA ≥ 0.1 ng/mL had lower odds (OR: 0.39, CI: 0.20–0.74) of undergoing salvage therapy. Among pN+ patients, 51% received adjuvant and 49% received salvage therapy. **Conclusions**: The management of high-risk prostate cancer remains varied. In our regional cohort, rates of salvage versus adjuvant therapy increased after publication of level-one evidence. Further work is warranted to better delineate who will most benefit from adjuvant versus early salvage therapy.

## Simple Summary

Recent high-quality evidence has shown that patients with certain high-risk features who undergo radical prostatectomy for cancer can safely defer subsequent radiation therapy until detectable rises in prostate-specific antigen (PSA) are found. However, questions remain regarding specific PSA criteria for initiating radiation therapy, and whether these findings can be applied to patients who are found to have lymph node metastases at the time of surgery. The aim of our retrospective study was to investigate real-world management patterns for these patients and identify factors that may influence choice of treatment pathway. The findings of this study can help to better determine who will most benefit from immediate versus deferred radiation therapy.

## 1. Introduction

Prostate cancer (CaP) remains the most diagnosed malignancy in men, and the incidence of high-grade disease at diagnosis is increasing [1,2]. High-risk features on final pathology after radical prostatectomy (RP), such as grade group (GG) 4 or 5 disease, positive surgical margins (PSM), and high tumor stage (T3b or higher) are associated with increased risk of recurrence, progression to metastatic disease, and mortality [3,4]. For high-risk patients, prior practice has included the use of planned adjuvant radiotherapy (RT). Recent landmark clinical trials [5,6,7] and prospectively planned meta-analyses [8] have shown patients with high-risk features who undergo radical prostatectomy and subsequently undergo early salvage RT after biochemical recurrence (BCR) have non-inferior oncologic outcomes, compared to those treated with planned adjuvant RT after RP. These level-one data have been incorporated into guidelines, with the American Urological Association (AUA) now strongly recommending against routinely recommending adjuvant RT after RP to mitigate harms associated with planned RT [9]. At this time, the European Association of Urology (EAU) still recommends adjuvant RT for these patients [10].

Nonetheless, ambiguity remains regarding the ideal prostate specific antigen (PSA) level to trigger early salvage RT in high-risk patients. Clinical trials contributing to guideline statements have utilized various PSA cutoff values. RAVES specified a PSA threshold of 0.2 ng/mL [5], GETUG-AFU 17 required 0.2 ng/mL and rising [6], while RADICALS-RT used a value of PSA >0.1 ng/mL obtained after two consecutive rising values or simply three consecutive rising values [7]. Currently, the AUA provide a conditional recommendation that providers may offer salvage RT for PSA < 0.2 ng/mL in this population, but specify they should provide salvage RT for PSA ≤ 0.5 ng/mL [11]. The EAU recommend starting salvage RT after two consecutive PSA increases rather than waiting to achieve a specific threshold [12].

Lymph node-positive disease discovered at the time of RP (pN+) in the absence of clinical nodal involvement is another high-risk feature which may warrant early RT. Previous work has shown that higher numbers of disease positive lymph nodes are associated with higher rates of disease recurrence [13,14]. However, retrospective evidence has shown a significant proportion of these patients remain alive without recurrence for 10 years post-RP, in the absence of secondary treatment [15]. Heterogeneity among pN+ patients may contribute to a lack of strong societal recommendations regarding treatment options for these patients, as current AUA and EAU guidelines remain equivocal [9,12].

Considering updated guidelines in the absence of clear PSA triggers, we sought to investigate real-world management patterns for patients with clinically localized CaP found to have high-risk features or positive lymph nodes after RP. We hypothesized that utilization of early salvage RT would increase after publication of the aforementioned landmark trials, but that overall management patterns would remain varied and driven by both patient-specific and institutional factors.

## 2. Materials and Methods

### 2.1. Study Cohort

Data from the Pennsylvania Urologic Regional Collaborative/Urologic Surgeons Comparing Outcomes Pursuing Excellence (PURC) database were utilized. The PURC database is a prospectively maintained quality improvement collaborative comprising 14 urology practices, including 169 urologists, in the Mid-Atlantic region. Practices in the PURC include private, community, and academic institutions that cover a wide range of communities, including urban and rural settings. Each participating practice site has obtained approval from their respective institutional review board, and informed consent was waived as the research involved no more than minimal risk to participants. Patients with nonmetastatic, clinically N0 adenocarcinoma of the prostate with at least one specified high-risk feature were included. High-risk features were pathologic stage T3 or T4 (pT3-4), Gleason score 7–10, PSM, preoperative PSA level ≥ 10 ng/mL, or pN+ disease identified at RP. Patients included underwent primary treatment with RP followed by secondary therapy between May 2015 (the first year of data abstraction into PURC) to January 2024 (most recent data available at the time of analysis). This study followed the Strengthening the Reporting of Observational Studies in Epidemiology (STROBE) reporting guidelines [16].

### 2.2. Study Outcomes

The primary outcome was to assess patient and institution-specific factors associated with the timing of secondary radiotherapy (adjuvant vs. salvage) for those with CaP with high-risk features. Secondary treatment is captured categorically as RT, androgen deprivation therapy (ADT), or chemotherapy, and entered discretely as either adjuvant or salvage therapy within the PURC database. Demographic variables included age, race, ethnicity, insurance, marital status, family history of CaP, and comorbidities. Clinicopathological variables of interest included year of RP, year of initiating secondary therapy, pathological T stage (pT), pathologic lymph node positivity (pN), Gleason score, margin positivity, pre- and postoperative PSA, pre-secondary treatment PSA (defined as PSA immediately prior to receiving secondary therapy), days from RP to receipt of secondary therapy, and secondary treatment type.

### 2.3. Statistical Analysis

Demographic and clinicopathological data were reported as frequencies and percentages or medians with interquartile range (IQR). Categorical and continuous variables were evaluated using Pearson’s chi-square tests and Student’s *t*-tests, respectively. Multivariable logistic regression was used to determine the association between study variables and the timing of secondary therapy (adjuvant vs. salvage) while controlling for potential confounders, including secondary treatment type, with results reported in terms of odds ratio (OR) and 95% confidence interval (CI). All analyses were conducted using Stata version 18.0 (StataCorp LLC, College Station, TX, USA) with statistical significance set at *p* value < 0.05.

## 3. Results

From the PURC dataset, 605 patients met the inclusion criteria between May 2015 and June 2024. Of those, 230 (38.0%) received adjuvant therapy and 375 (62.0%) received salvage therapy. When comparing the groups’ demographics (Table 1), patients who received adjuvant therapy were associated with private insurance (35.2% vs. 20.8%; *p* < 0.001) or Medicare/Medicaid (28.7% vs. 22.1%; *p* < 0.001). The majority of patients were treated at either facility A (30.2%) or facility C (37.2%). Those at facility A more commonly received adjuvant therapy (47.0% vs. 20.0%; *p* < 0.001), while those at facility C less frequently received adjuvant therapy (11.3% vs. 53.1%; *p* < 0.001). No significant differences were observed between the two groups in any other demographic characteristics.

**Figure 1:**
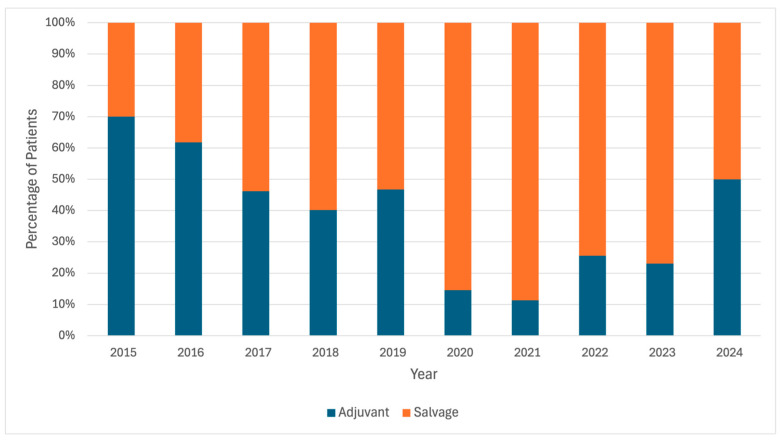
Frequency of secondary treatment over time.

Summarizing the high-risk features of the study cohort (Table 2): 17.5% had pN+ disease, 74.2% had pT3-4, 95.5% had a Gleason score of 7 or higher, 55.0% had a positive surgical margin, and 40.7% had a preoperative PSA level ≥ 10 ng/mL. The overall median initial post-RP PSA was 0.09 ng/mL (IQR: 0.09–0.40 ng/mL). Between the adjuvant and salvage groups, there was no significant difference in preoperative PSA (*p* = 0.46) or postoperative PSA immediately following RP (*p* = 0.45). However, patients with a postoperative PSA ≥ 0.1 ng/mL were associated with receiving adjuvant therapy (57.4% vs. 43.5%; *p* < 0.001). The adjuvant group also had a lower PSA level prior to starting secondary treatment (0.24 ng/mL vs. 0.27 ng/mL; *p* = 0.02). A significantly higher percentage of patients received salvage therapy (38.9%) compared to adjuvant therapy (13.5%) in or after 2020 (*p* < 0.001). An increase in salvage therapy is also seen after 2020 when stratified by each year (Figure 1).

Multivariable regression analysis was conducted to evaluate factors associated with the receipt of adjuvant versus salvage therapy (Table 3). Factors associated with higher odds of receiving salvage therapy included preoperative PSA ≥ 10 ng/mL (OR: 2.15, CI: 1.31–3.53), treatment in or after 2020 (OR: 3.41, CI: 1.75–6.66), and secondary treatment with RT (OR: 2.75, CI: 1.52–5.00). Patients at facility C also had a higher odds of receiving salvage therapy (OR: 5.26, CI: 1.73–15.93). Patients with a persistent postoperative PSA ≥ 0.1 ng/mL had lower odds of receiving salvage therapy (OR: 0.39, CI: 0.20–0.74).

Evaluating the 375 patients who received salvage therapy (Table 4), the median days from RP to starting salvage therapy was 350 days (IQR: 185–658 days). The majority of patients (91.5%) received salvage therapy after rising PSA values. Most patients (70.3%) also had a PSA ≥ 0.2 ng/mL prior to initiating salvage therapy. Further investigation of the salvage group was conducted by comparing those who initiated salvage therapy prior to 2020 (61.1%) versus those who started salvage in or after 2020 (38.9%). Between the two groups, the median days from RP to starting salvage therapy increased after 2020 (488 days vs. 281 days; *p* < 0.001). Additionally, there was an increase in patients who started salvage therapy with a rising versus static PSA (95.2% vs. 89.1%; *p* = 0.04).

Among the 106 patients with pN+ disease identified at RP (Table 5), 50.9% received adjuvant therapy and 49.1% received salvage therapy. The median days from RP to adjuvant therapy was 102 days, while the median days from RP to salvage therapy was 150 days (*p* < 0.001). Of the pN+ disease patients that received adjuvant therapy, 69.0% had a pre-secondary treatment PSA ≥ 0.2 ng/mL; while 78.7% of pN+ disease patients that received salvage therapy had both pre-secondary treatment PSA ≥ 0.2 ng/mL and rising PSA values (*p* < 0.001).

## 4. Discussion

In our study, we leveraged the use of a multi-institutional cohort to examine the utilization of adjuvant versus salvage therapy in high-risk prostate cancer patients post-radical prostatectomy. We confirmed our hypothesis that use of early salvage therapy would increase after landmark clinical trial publication. This is significant when taken in the greater context that the majority of patients in our cohort underwent surveillance with early salvage therapy, even prior to landmark trial publication and subsequent guideline amendments. The explicit reasoning behind clinical decision making in this cohort is unattainable from the PURC database. However, a possible explanation for the apparent adoption of salvage therapy earlier than expected is that debate surrounding its use began long before the initiation of the landmark trials that led to the guideline changes [17]. It is possible that the accumulation of non-level one evidence had already influenced the decision making of many clinicians treating patients in this cohort, and indeed previous work suggests that the use of adjuvant RT was already declining during this time [18].

Additionally, we confirmed our hypothesis that management patterns for our high-risk cohort remained varied and driven by patient- and institution-specific factors. Though the majority of our cohort were treated at two institutions, they differed significantly in proportion of adjuvant versus salvage therapy. Though PURC lacks details about practice setting and other institution-level data, this suggests that receipt of secondary therapy may be influenced by factors beyond the individual patient, consistent with the existing literature [19]. Regarding patient-specific factors, we further identified characteristics such as elevated preoperative PSA (≥10 ng/mL), secondary treatment beginning in or after 2020, and secondary treatment with RT as significant factors predicting the receipt of salvage therapy. The findings of elevated preoperative PSA as a significant predictor of salvage therapy is somewhat surprising, as this characteristic is traditionally associated with increased risk for BCR and therefore would be expected to predict adjuvant therapy. Given the variety of factors that can cause elevations in PSA, it is possible that this metric is less heavily weighted during clinical decision making by the providers captured in this study. We also found that elevated postoperative PSA (≥0.1 ng/mL) was a significant factor predicting against the receipt of salvage therapy. This follows the existing literature, which identified detectable postoperative PSA to be a poor prognostic indicator. Though characteristics such as Gleason score, pT staging, pN+ status, and PSM differed between cohorts, none were found to be significant predictors in our model. The reasoning for this is not clear but likely reflects the inherent heterogeneity in the constructed high-risk cohort. We also found a significant difference in receipt of adjuvant versus salvage therapy based on type of patient insurance. This may represent a problem with data abstraction in the PURC dataset, as significantly more patients who underwent salvage therapy were categorized as having “other” or “unknown” insurance providers. Regardless, one retrospective, cohort study found that private insurance was a significant positive predictor of patients with clinically insignificant CaP receiving RP [20], suggesting that insurance could play a role in treatment pathways. However, there appears to be a dearth of literature specifically examining the influence of insurance type on post-RP treatment selection, so this may be a worthwhile question for future investigations.

The exact PSA level used to initiate early salvage therapy remains varied. The AUA suggest a value of 0.2 ng/mL, above which treatment should be initiated and below which treatment may be initiated based on other clinical factors [11]. The EAU recommend beginning treatment after two consecutive increases in PSA [12]. In the landmark trials, the investigators in RAVES utilized a PSA threshold of 0.2 ng/mL [5], while those in GETUG-AFU 17 used 0.2 ng/mL and rising [6], and RADICALS-RT studied a PSA greater than 0.1 ng/mL following two consecutive rising PSA values or three consecutive rising values [7]. In the present study, nearly all patients in the salvage cohort had secondary therapy initiated in the setting of a rising PSA; however, a notable limitation of the database was that we were unable to ascertain exactly how many rising values were recorded. The specific PSA at which therapy was initiated in this cohort was most commonly ≥ 0.2 ng/mL, with < 0.1 ng/mL being extremely uncommon and an overall median value of 0.27 ng/mL. These results seem to more closely follow the EAU recommendations and/or the RAVES/GETUG-AFU 17 criteria. In the absence of level one evidence showing a PSA cutoff of 0.1 mg/mL to be superior to 0.2 ng/mL, it is reasonable that clinicians may choose the latter as it may delay patients from experiencing the negative effects of secondary therapies for a longer period of time.

We noted a nonsignificant decrease in median PSA values for initiating salvage therapy pre- and post-2020 with the publication of the trials. Though this result did not reach statistical significance, it could signify an adaptation of earlier salvage therapy by clinicians. Interestingly, though the PSA value prior to initiation of salvage therapy did not significantly change, we noted the emergence of a significant temporal delay as the median time from RP to salvage therapy increased from 281 to 488 days post-2020. A possible reason for this increase in time to salvage therapy is that patients who would previously have been treated with adjuvant therapy were now being observed and who did not require salvage treatment until much later if indeed they required additional treatment at all. This trend is likely also influenced by the increasing emphasis on monitoring PSA trends rather than initiating therapy at the first detectable PSA [11]. This finding is consistent with the amendments to the AUA guidelines that favor deferring initiation of therapy upon PSA elevation.

PSA doubling time (PSADT) is a valuable prognostic indicator used to assess disease progression post-RP and provide insight on a patient’s likelihood to develop metastases as well as predicting a favorable response to salvage RT [21,22]. There has been some debate regarding the methodology utilized to calculate PSADT with studies using 3 months and 6 months to reveal high-risk patients. Rapid rises in PSA over a shorter interval was shown to reflect a more aggressive form of disease that necessitated earlier salvage therapy intervention [23]. There are many clinical parameters that will impact a patients’ response to RT with studies indicating improved outcomes when given at low PSA levels in cases of poorly differentiated cancer and short PSADT [24]. This underscores the merit of utilizing PSA kinetics to facilitate patients receiving timely and appropriate treatment. We also sought to characterize a cohort of pN+ patients and found highly variable management, consistent with the existing literature [19]. In our cohort, patients were split approximately evenly between adjuvant and salvage pathways. This is reflective of the overall heterogeneity of a pN+ disease state [15] and the lack of strong guideline statements regarding management of these patients [9,12]. Importantly, a significant limitation to our study is that we were unable to ascertain the exact number of positive nodes, a characteristic that has been shown to be predictive of several oncologic outcomes [13,14]. Though we performed no formal comparison, pN+ patients who went on to receive salvage therapy appeared to do so closer to the time of RP compared to the overall cohort. This is likely reflective of the existing principle that node positivity places an individual at higher risk for BCR and disease progression. To date, only one randomized clinical trial (ECOG 3886) has been published which investigated the management of pN+ patients [25]. 100 pN+ patients were randomized to immediate, lifelong ADT versus observation with salvage ADT. Adjuvant patients were shown to have improved overall survival; however, BCR was not sufficient to initiate ADT in the observation cohort and only one patient received salvage RT with ADT. Several retrospective studies have supported the role of RT in these patients. One study of 270 patients receiving secondary RT treatment with or without ADT noted favorable biochemical progression-free survival, with increasing success at low pre-RT PSA levels (83% at <0.1 ng/mL, 76% at 0.1–<0.5 ng/mL, 60% at 0.5–2 ng/mL) [26]. Other studies have similarly substantiated the utility of RT as either a monotherapy or in combination with ADT [27]. Accordingly, we noted a nonsignificant trend towards RT compared to ADT in the salvage pN+ cohort. Ultrasensitive PSA (uPSA) utilization may also represent an opportunity for a more robust risk stratification post-RP. One study involving 188 pN+ patients suggested that uPSA could be used to allow observation with early salvage therapy [28]. This may represent a valuable tool to inform clinical decision making in this higher risk population.

As with any study, ours is not without its limitations. This study is inclusive of all adjuvant and salvage therapy patients, and is not strictly limited to those receiving RT. A greater proportion of patients in our study received secondary treatment prior to 2020, potentially skewing our results. As with all database-driven studies, there is always risk of misclassification among reporters during data abstraction. Several limitations are inherent to the PURC database. Due to the nature of data recording, patients who are currently undergoing observation with planned early salvage therapy if required are not captured in our study cohort. This is similarly true for pN+ patients that have, to date, received no secondary treatment. Participation in PURC is voluntary and regional; thus, our data may not be broadly generalizable. Additionally, despite the robustness of the database, we can never wholly understand the complete clinical context of the individual patient that led to certain decisions. Discrete categorization of secondary therapies may introduce bias due to institutional differences in defining adjuvant versus salvage therapy. Additional limitations include lack of data regarding the extent of the lymph node dissection performed, the number of positive nodes in the pN+ population, and imaging findings after RP (including emerging modalities such as PSMA PET CT/MRI findings). Studies have shown these factors affect patient management and are predictors of biochemical recurrence and cancer specific survival [29,30,31]. Additionally, the lack of data on genomic risk scores (Decipher, Select MDx, etc.) limits further analysis on the utilization of genomic classifiers in patient management [32]. We also lack data on practice setting. This may represent a significant confounding variable, as has been suggested in other similar studies [19].

## 5. Conclusions

This retrospective study reflects real-world management patterns for patients with high-risk prostate cancer undergoing radical prostatectomy, with a focus on the decision-making process between immediate versus deferred radiation therapy. The management of high-risk CaP after RP remains varied, though clinicians are increasingly moving towards observation with early salvage therapy as the preferred treatment pathway. Notably, receipt of secondary therapy in or after 2020 was a significant predictor of clinicians opting for salvage over adjuvant therapy.

The growing preference for observation with salvage therapy reflects an evolving understanding shaped by recent level-one evidence. However, even after publication of landmark trials, heterogeneity in clinical practice persists. The variability in postoperative management highlights a key clinical challenge: our current risk stratification tools remain imperfect. As a result, clinicians must balance the risk of overtreatment with the potential consequences of undertreatment—aiming to administer therapy early enough to benefit those who need it, while avoiding unnecessary toxicity in those who do not.

While a PSA threshold of <0.2 ng/mL is increasingly accepted for initiating salvage therapy, the cutoff’s applicability to pN+ patients remains uncertain. This subgroup may benefit from use of ultrasensitive PSA assays and prospective evaluation of tailored PSA thresholds for initiating salvage therapy. Emerging diagnostic modalities may also provide further insight into understanding which patients will benefit from adjuvant therapy. Furthermore, the management of pN+ patients would greatly benefit from prospective, randomized clinical trials to further elucidate optimal treatment pathways.

## Figures and Tables

**Table 1:** Demographics of study cohort.

Variable	Total (*n* = 605)	Adjuvant (*n* = 230)	Salvage (*n* = 375)	*p* Value
Age Range (years)				0.96
49 or under	6 (1.0%)	3 (1.3%)	3 (0.8%)	
50–59	45 (7.4%)	17 (7.4%)	28 (7.5%)	
60–69	265 (43.8%)	101 (43.9%)	164 (43.7%)	
70–79	259 (42.8%)	99 (43.0%)	160 (42.7%)	
80–89	30 (5.0%)	10 (4.3%)	20 (5.3%)	
Race				0.14
African American	117 (19.3%)	51 (22.2%)	66 (17.6%)	
Asian	12 (2.0%)	7 (3.0%)	5 (1.3%)	
Caucasian	458 (75.7%)	163 (70.9%)	295 (78.7%)	
Hawaiian/Pacific Islander	1 (0.2%)	1 (0.4%)	0 (0%)	
Other/Unknown/Refused	17 (2.8%)	8 (3.5%)	9 (2.4%)	
Ethnicity				0.12
Non-Hispanic	586 (96.9%)	219 (95.2%)	367 (97.9%)	
Hispanic	14 (2.3%)	9 (3.9%)	5 (1.3%)	
Unknown/Refused	5 (0.8%)	2 (0.9%)	3 (0.8%)	
Family History of CaP				0.34
1st degree	121 (20.0%)	46 (20.0%)	75 (20.0%)	
2nd degree	37 (6.1%)	16 (7.0%)	21 (5.6%)	
Both	15 (2.5%)	4 (1.7%)	11 (2.9%)	
Positive, relation unknown	2 (0.3%)	0 (0%)	2 (0.5%)	
Unknown	43 (7.1%)	11 (4.8%)	32 (8.5%)	
None	387 (64.0%)	153 (66.5%)	234 (62.4%)	
Insurance				<0.001
Private	159 (26.3%)	81 (35.2%)	78 (20.8%)	
Medicare/Medicaid	149 (24.6%)	66 (28.7%)	83 (22.1%)	
Tricare/VA	8 (1.3%)	1 (0.4%)	7 (1.9%)	
Self-pay	5 (0.8%)	1 (0.4%)	4 (1.1%)	
Other	96 (15.9%)	12 (5.2%)	84 (22.4%)	
Unknown	188 (31.1%)	69 (30.0%)	119 (31.7%)	
Marital Status				0.31
Single	37 (6.1%)	19 (8.3%)	18 (4.8%)	
Married/Partnered	285 (47.1%)	110 (47.8%)	175 (46.7%)	
Separated/Divorced	24 (4.0%)	7 (3.0%)	17 (4.5%)	
Widowed	10 (1.7%)	5 (2.2%)	5 (1.3%)	
Unknown	249 (41.2%)	89 (38.7%)	160 (42.7%)	
Comorbidities				
Cerebrovascular Disease	31 (5.1%)	15 (6.5%)	16 (4.3%)	0.22
Chronic Pulmonary Disease	30 (5.0%)	10 (4.3%)	20 (5.3%)	0.59
Diabetes without Organ Damage	76 (12.6%)	30 (13.0%)	46 (12.3%)	0.78
Diabetes with Organ Damage	7 (1.2%)	2 (0.9%)	5 (1.3%)	0.60
Myocardial Infarction	18 (3.0%)	3 (1.3%)	15 (4.0%)	0.06
Peripheral Vascular Disease	10 (1.7%)	5 (2.2%)	5 (1.3%)	0.43
Facility				<0.001
A	183 (30.2%)	108 (47.0%)	75 (20.0%)	
B	12 (2.0%)	9 (3.9%)	3 (0.8%)	
C	225 (37.2%)	26 (11.3%)	199 (53.1%)	
D	29 (4.8%)	21 (9.1%)	8 (2.1%)	
E	4 (0.7%)	3 (1.3%)	1 (0.3%)	
F	11 (1.8%)	8 (3.5%)	3 (0.8%)	
G	34 (5.6%)	21 (9.1%)	13 (3.5%)	
H	40 (6.6%)	14 (6.1%)	26 (6.9%)	
I	66 (10.9%)	19 (8.3%)	47 (12.5%)	
J	1 (0.2%)	1 (0.4%)	0 (0.0%)	

Abbreviations: CaP, prostate cancer; VA, veterans affairs.

**Table 2:** Clinicopathological characteristics of study cohort.

Variable	Total (*n* = 605)	Adjuvant (*n* = 230)	Salvage (*n* = 375)	*p* Value
Year of RP				0.03
2015	57 (9.4%)	25 (10.9%)	32 (8.5%)	
2016	113 (18.7%)	44 (19.1%)	69 (18.4%)	
2017	165 (27.3%)	57 (24.8%)	108 (28.8%)	
2018	106 (17.5%)	51 (22.2%)	55 (14.7%)	
2019	78 (12.9%)	27 (11.7%)	51 (13.6%)	
2020	41 (6.8%)	6 (2.6%)	35 (9.3%)	
2021	28 (4.6%)	12 (5.2%)	16 (4.3%)	
2022	15 (2.5%)	7 (3.0%)	8 (2.1%)	
2023	2 (0.3%)	1 (0.4%)	1 (0.3%)	
Year of RP (pre/post-2020)				0.11
<2020	519 (85.8%)	204 (88.7%)	315 (84.0%)	
≥2020	86 (14.2%)	26 (11.3%)	60 (16.0%)	
pT				<0.001
T2	156 (25.8%)	36 (15.7%)	120 (32.0%)	
T3a	196 (32.4%)	83 (36.1%)	113 (30.1%)	
T3b	251 (41.5%)	111 (48.3%)	140 (37.3%)	
T4	2 (0.3%)	0 (0%)	2 (0.5%)	
pN				0.003
N0	499 (82.5%)	176 (76.5%)	323 (86.1%)	
N1	106 (17.5%)	54 (23.5%)	52 (13.9%)	
Gleason Score				0.01
6	3 (0.5%)	2 (0.9%)	1 (0.3%)	
7	291 (49.3%)	95 (42.2%)	196 (53.7%)	
8	103 (17.5%)	37 (16.4%)	66 (18.1%)	
9	187 (31.7%)	87 (38.7%)	100 (27.4%)	
10	6 (1.0%)	4 (1.8%)	2 (0.5%)	
Surgical Margin				0.003
Negative	272 (45.0%)	86 (37.4%)	186 (49.7%)	
Positive	332 (55.0%)	144 (62.6%)	188 (50.3%)	
Pre-op PSA (ng/mL)	8.20 (5.40–14.18)	7.90 (5.19–14.54)	8.31 (5.50–14.01)	0.46
Pre-op PSA Range (ng/mL)				0.97
PSA < 10	340 (59.3%)	126 (59.4%)	214 (59.3%)	
PSA ≥ 10	233 (40.7%)	86 (40.6%)	147 (40.7%)	
Post-op PSA (ng/mL)	0.09 (0.09–0.40)	0.10 (0.06–0.55)	0.09 (0.09–0.30)	0.45
Post-op PSA Range (ng/mL)				<0.001
PSA < 0.1	310 (51.2%)	98 (42.6%)	212 (56.5%)	
PSA ≥ 0.1	295 (48.8%)	132 (57.4%)	163 (43.5%)	
Pre-Secondary Tx PSA (ng/mL)	0.26 (0.12–0.70)	0.24 (0.09–0.80)	0.27 (0.16–0.68)	0.02
Secondary Tx Type				<0.001
ADT	162 (26.8%)	82 (35.7%)	80 (21.3%)	
Chemotherapy	5 (0.8%)	0 (0%)	5 (1.3%)	
EBRT	438 (72.4%)	148 (64.3%)	290 (77.3%)	
Year Secondary Tx Initiated				<0.001
2015	10 (1.7%)	7 (3.0%)	3 (0.8%)	
2016	55 (9.1%)	34 (14.8%)	21 (5.6%)	
2017	104 (17.2%)	48 (20.9%)	56 (14.9%)	
2018	167 (27.6%)	67 (29.1%)	100 (26.7%)	
2019	92 (15.2%)	43 (18.7%)	49 (13.1%)	
2020	62 (10.2%)	9 (3.9%)	53 (14.1%)	
2021	53 (8.8%)	6 (2.6%)	47 (12.5%)	
2022	47 (7.8%)	12 (5.2%)	35 (9.3%)	
2023	13 (2.1%)	3 (1.3%)	10 (2.7%)	
2024	2 (0.3%)	1 (0.4%)	1 (0.3%)	
Secondary Tx Initiated Before/After 2020				<0.001
<2020	428 (70.7%)	199 (86.5%)	229 (61.1%)	
≥2020	177 (29.3%)	31 (13.5%)	146 (38.9%)	
Days From RP to Secondary Tx	243 (140–470)	166 (113–253)	350 (185–658)	<0.001

Abbreviations: ADT, androgen deprivation therapy; EBRT, external beam radiation therapy; pN, pathologic N stage; PSA, prostate specific antigen; pT, pathologic T stage; RP, radical prostatectomy; Tx, treatment.

**Table 3:** Multivariable analysis of patient factors associated with receipt of salvage therapy.

Variable	OR	*p* Value	CI
Insurance			
Private (ref.)	1	–	–
Medicare/Medicaid	0.87	0.66	0.46–1.62
Tricare/VA	5.39	0.16	0.51–56.41
Self-pay	0.13	0.11	0.01–1.54
Other	0.92	0.87	0.33–2.54
Unknown	1.12	0.73	0.58–2.17
Facility			
A	0.74	0.53	0.28–1.92
B	0.41	0.31	0.07–2.29
C	5.26	0.003	1.73–15.93
D	0.47	0.27	0.13–1.78
E	1.81	0.73	0.06–51.97
F	0.98	0.98	0.17–5.53
G	0.75	0.64	0.22–2.57
H (ref.)	1	–	–
I	2.63	0.09	0.86–7.97
J	1	–	–
pT			
T2 (ref.)	1	–	–
T3 or T4	1.06	0.81	0.65–1.73
pN			
N0 (ref.)	1	–	–
N+	0.78	0.45	0.41–1.48
Gleason Score	0.95	0.69	0.73–1.23
Margin			
Negative (ref.)	1	–	–
Positive	0.85	0.52	0.52–1.39
Pre-op PSA Range			
<10 ng/mL (ref.)	1	–	–
≥10 ng/mL	2.15	0.002	1.31–3.53
Post-op PSA Range			
<0.1 ng/mL (ref.)	1	–	–
≥0.1 ng/mL	0.39	0.004	0.20–0.74
Pre-Secondary Tx PSA Range (ng/mL)			
PSA < 0.1	0.2	<0.001	0.09–0.44
0.1 ≤ PSA < 0.2 (ref.)	1	–	–
PSA ≥ 0.2	1.48	0.26	0.75–2.93
Secondary Tx Initiated Before/After 2020			
<2020 (ref.)	1	–	–
≥2020	3.41	<0.001	1.75–6.66
Secondary Tx Type			
ADT (ref.)	1	–	–
Chemotherapy	1	–	–
EBRT	2.75	0.001	1.52–5.00

Abbreviations: ADT, androgen deprivation therapy; EBRT, external beam radiation therapy; pN, pathologic N stage; PSA, prostate specific antigen; pT, pathologic T stage; Tx, treatment; VA, veterans affairs.

**Table 4:** Factors of salvage therapy group before vs. after 2020.

Variable	Total Salvage (*n* = 375)	<2020 (*n* = 229)	≥2020 (*n* = 146)	*p* Value
Year of RP				<0.001
2015	32 (8.5%)	30 (13.1%)	2 (1.4%)	
2016	69 (18.4%)	65 (28.4%)	4 (2.7%)	
2017	108 (28.8%)	89 (38.9%)	19 (13.0%)	
2018	55 (14.7%)	38 (16.6%)	17 (11.6%)	
2019	51 (13.6%)	7 (3.1%)	44 (30.1%)	
2020	35 (9.3%)	0 (0%)	35 (24.0%)	
2021	16 (4.3%)	0 (0%)	16 (11.0%)	
2022	8 (2.1%)	0 (0%)	8 (5.5%)	
2023	1 (0.3%)	0 (0%)	1 (0.7%)	
Year of RP (pre/post-2020)				<0.001
<2020	315 (84.0%)	229 (100%)	86 (58.9%)	
≥2020	60 (16.0%)	0 (0%)	60 (41.1%)	
Days From RP to Secondary Tx	350 (185–658)	281 (170–489)	488 (221–1064)	<0.001
Pre-Secondary Tx PSA (ng/mL)	0.27 (0.16–0.68)	0.30 (0.16–0.77)	0.22 (0.16–0.49)	0.26
Rising PSA				0.04
No	32 (8.5%)	25 (10.9%)	7 (4.8%)	
Yes	343 (91.5%)	204 (89.1%)	139 (95.2%)	
Pre-secondary Tx PSA Range (ng/mL)				0.73
PSA < 0.1	34 (9.3%)	22 (9.9%)	12 (8.3%)	
0.1 ≤ PSA < 0.2	75 (20.4%)	43 (19.3%)	32 (22.2%)	
PSA ≥ 0.2	258 (70.3%)	158 (70.9%)	100 (69.4%)	
Pre-secondary Tx PSA Range (ng/mL) and Rising				0.32
PSA < 0.1, not rising	8 (2.2%)	6 (2.7%)	2 (1.4%)	
0.1 ≤ PSA < 0.2, not rising	5 (1.4%)	5 (2.2%)	0 (0.0%)	
PSA ≥ 0.2, not rising	17 (4.6%)	12 (5.4%)	5 (3.5%)	
PSA < 0.1 and rising	26 (7.1%)	16 (7.2%)	10 (6.9%)	
0.1 ≤ PSA < 0.2 and rising	70 (19.1%)	38 (17.0%)	32 (22.2%)	
PSA ≥ 0.2 and rising	241 (65.7%)	146 (65.5%)	95 (66.0%)	

Abbreviations: PSA, prostate-specific antigen; RP, radical prostatectomy; Tx, treatment.

**Table 5:** Factors of pN+ group by adjuvant vs. salvage therapy.

Variable	Total pN+ (*n* = 106)	Adjuvant (*n* = 54)	Salvage (*n* = 52)	*p* Value
Days From RP to Secondary Tx	126 (77–202)	102 (51–149)	150 (108–248)	<0.001
Secondary Tx Type				0.06
ADT	60 (56.6%)	36 (66.7%)	24 (46.2%)	
Chemotherapy	2 (1.9%)	0 (0%)	2 (3.8%)	
EBRT	44 (41.5%)	18 (33.3%)	26 (50.0%)	
Gleason Score				0.28
7	29 (28.2%)	12 (23.1%)	17 (33.3%)	
8	19 (18.4%)	10 (19.2%)	9 (17.6%)	
9	53 (51.5%)	30 (57.7%)	23 (45.1%)	
10	2 (1.9%)	0 (0%)	2 (3.9%)	
Surgical Margin				0.78
Negative	34 (32.1%)	18 (33.3%)	16 (30.8%)	
Positive	72 (67.9%)	36 (66.7%)	36 (69.2%)	
pT				0.30
T2	10 (9.4%)	3 (5.6%)	7 (13.5%)	
T3a	20 (18.9%)	12 (22.2%)	8 (15.4%)	
T3b	76 (71.7%)	39 (72.2%)	37 (71.2%)	
Pre-op PSA (ng/mL)	11.47 (6.90–23.04)	8.70 (5.30–18.20)	13.70 (9.26–26.35)	0.01
Post-op PSA (ng/mL)	0.50 (0.10–2.16)	0.40 (0.09–1.34)	0.59 (0.18–5.95)	0.12
Pre-Secondary Tx PSA (ng/mL)	0.67 (0.20–3.12)	0.69 (0.09–2.40)	0.60 (0.20–6.60)	0.26
Rising PSA				<0.001
No	58 (54.7%)	54 (100%)	4 (7.7%)	
Yes	48 (45.3%)	0 (0%)	48 (92.3%)	
Pre-secondary Tx PSA Range (ng/mL)				0.06
PSA < 0.1	15 (16.9%)	11 (26.2%)	4 (8.5%)	
0.1 ≤ PSA < 0.2	7 (7.9%)	2 (4.8%)	5 (10.6%)	
PSA ≥ 0.2	67 (75.3%)	29 (69.0%)	38 (80.9%)	
Pre-secondary Tx PSA Range (ng/mL) and Rising				<0.001
PSA < 0.1, not rising	12 (13.5%)	11 (26.2%)	1 (2.1%)	
0.1 ≤ PSA < 0.2, not rising	3 (3.4%)	2 (4.8%)	1 (2.1%)	
PSA ≥ 0.2, not rising	30 (33.7%)	29 (69.0%)	1 (2.1%)	
PSA < 0.1 and rising	3 (3.4%)	0 (0.0%)	3 (6.4%)	
0.1 ≤ PSA < 0.2 and rising	4 (4.5%)	0 (0.0%)	4 (8.5%)	
PSA ≥ 0.2 and rising	37 (41.6%)	0 (0.0%)	37 (78.7%)	

Abbreviations: ADT, androgen deprivation therapy; EBRT, external beam radiation therapy; pN, pathologic N stage; PSA, prostate specific antigen; pT, pathologic T stage; Tx, treatment.

## Data Availability

Data were provided with permission from the Pennsylvania Urologic Regional Collaborative (PURC) participating urology practices. PURC is a quality improvement initiative led by the Health Care Improvement Foundation which brings urology practices together in a physician-led, data-sharing and improvement collaborative aimed at advancing the quality of diagnosis and care for men with prostate cancer.
